# Stochastic coupled simulation of strong motion and tsunami for the 2011 Tohoku, Japan earthquake

**DOI:** 10.1007/s00477-016-1352-1

**Published:** 2016-11-12

**Authors:** Katsuichiro Goda, Crescenzo Petrone, Raffaele De Risi, Tiziana Rossetto

**Affiliations:** 1 Department of Civil Engineering, Queen’s School of Engineering, University of Bristol, Bristol, UK; 2 Department of Civil, Environmental & Geomatic Engineering, University College London, London, UK

**Keywords:** Earthquake source modeling, Stochastic finite-fault ground-motion simulation, Stochastic tsunami simulation, 2011 Tohoku earthquake

## Abstract

This study conducts coupled simulation of strong motion and tsunami using stochastically generated earthquake source models. It is focused upon the 2011 Tohoku, Japan earthquake. The ground motion time-histories are simulated using the multiple-event stochastic finite-fault method, which takes into account multiple local rupture processes in strong motion generation areas. For tsunami simulation, multiple realizations of wave profiles are generated by evaluating nonlinear shallow water equations with run-up. Key objectives of this research are: (i) to investigate the sensitivity of strong motion and tsunami hazard parameters to asperities and strong motion generation areas, and (ii) to quantify the spatial variability and dependency of strong motion and tsunami predictions due to common earthquake sources. The investigations provide valuable insights in understanding the temporal and spatial impact of cascading earthquake hazards. Importantly, the study also develops an integrated strong motion and tsunami simulator, which is capable of capturing earthquake source uncertainty. Such an advanced numerical tool is necessary for assessing the performance of buildings and infrastructure that are subjected to cascading earthquake–tsunami hazards.

## Introduction

Large subduction earthquakes, which occur at plate boundaries globally (Kagan 2017), not only generate intense ground shaking but also trigger massive tsunami and geo-hazards. Recent major earthquakes, such as the 2004 Sumatra, Indonesia earthquake (Murata et al. 2010) and the 2011 Tohoku, Japan earthquake (Fraser et al. 2013; Goda et al. 2013), have shown that both primary and secondary hazards result in catastrophic consequences. Damage and loss caused by secondary hazards occasionally exceed those by primary hazards (Daniell et al. 2011), and thus all relevant hazards should be incorporated in earthquake disaster management. Moreover, consideration of cascading earthquake-induced hazards is vital in designing coastal structures and infrastructure (Federal Emergency Management Agency 2008). To model cascading strong motion-tsunami hazards, Maeda et al. (2013) developed a novel computational method for parallel simulation of seismic and tsunami waves. However, due to its high computational cost, numerous runs to assess the uncertainty of the earthquake–tsunami hazard simulations may not be practically feasible. Moreover, the method requires the further development of simulating inland tsunami inundation processes accurately. Therefore, at present, there is no viable computational tool that can be used to generate strong motion-tsunami load sequences by taking into account the physical rupture processes and the uncertainty of mega-thrust subduction earthquakes. Consequently, the current earthquake–tsunami hazard and risk assessment methodology is unable to evaluate the compounding effects of such cascading hazards on coastal structures (e.g. vertical evacuation buildings).

The fault rupture of mega-thrust subduction earthquakes is complex and has a significant influence on strong motion and tsunami (e.g. Yokota et al. 2011; Kurahashi and Iikura 2013). Characterizing earthquake rupture processes is challenging as they are governed by seismotectonic settings, frictional properties and pre-rupture stress conditions of a fault that are largely unknown. Inference of the space–time evolution of earthquake rupture can be conducted using source inversion analysis. Comparison of numerous inversion studies for the 2011 Tohoku earthquake indicated that published earthquake source models vary significantly in terms of fault geometry and spatial slip distribution (Goda et al. 2014; Razafindrakoto et al. 2015). As a common feature, strong motion-based source models have relatively small fault segments at deep locations (typically 25–40 km), which radiate intense high-frequency seismic waves (e.g. Asano and Iwata 2012; Kurahashi and Iikura 2013). These segments are referred to as strong motion generation areas (SMGA), and are physically related to fault rupture with large slip velocity or high stress drop (Irikura and Miyake 2011). On the other hand, tsunami-based source models produced source images that have large slip concentrations in shallow fault segments (less than 10 km deep) along the Japan Trench (e.g. Gusman et al. 2012; Satake et al. 2013), which characterize low-frequency components of the observed geophysical data. The area having large slip concentrations is often referred to as asperity. The source models based on teleseismic and geodetic data had a tendency to estimate asperities near the epicenter at intermediate depths (typically 10–25 km; e.g. Ammon et al. 2011; Shao et al. 2011). Currently, a unified theory that explains frequency-dependent earthquake rupture processes has not been fully developed for predictions of strong motion and tsunami for large subduction earthquakes. The linkages between the high-frequency and low-frequency rupture processes can be specified via empirical rules and scaling laws, such as the seismic moment ratio of segments and the area ratio of SMGAs to the total fault plane (Irikura and Miyake 2011; Morikawa et al. 2011).

This study carries out coupled simulation of strong motion and tsunami using stochastic earthquake source models. It focuses on the 2011 Tohoku earthquake, for which various shaking and tsunami data are available for retrospective validation. The analytical framework for the stochastic coupled simulation considers uncertainties associated with the rupture process explicitly. A stochastic synthesis method, used for the source modeling of background fault rupture, is based on the spectral representation of slip heterogeneity (Mai and Beroza 2002; Goda et al. 2014), and generates random fields that have geophysically realistic spatial slip distributions as revealed in inverted source models. Moreover, SMGAs that may be located at different places from asperities (Kurahashi and Iikura 2013) are incorporated in the rupture models. The ground motion time-histories are simulated using a multiple-event stochastic finite-fault (SFF) method (Ghofrani et al. 2013). It can generate time-histories with multiple-shock features due to SMGAs, in addition to the background fault rupture. The background fault rupture governs low-frequency components of the synthesized ground motion time-histories, whereas the SMGAs mainly control high-frequency components. In assessing the variability of strong motion simulations, parameters of the multiple-event SFF model (e.g. magnitude, stress drop and geometry of the background fault and SMGAs) are varied over certain ranges that are deemed as reasonable in light of current knowledge in geophysics and seismology. For tsunami, multiple realizations of wave height time-histories due to stochastic slip distributions are generated by evaluating nonlinear shallow water equations. The novelty of this research is consideration of common physical rupture processes in stochastic strong motion and tsunami simulations, facilitating the assessment of the dependency between shaking and tsunami hazard parameters and the sensitivity analysis of the hazard parameters to uncertain features of asperities and SMGAs. Such an advanced numerical tool is necessary for assessing the performance of buildings and coastal infrastructure that are subjected to large subduction earthquakes.

The paper is organized as follows. Section 2 presents an overview of the analytical framework for the stochastic coupled simulation of strong motion and tsunami by focusing on a common earthquake rupture process. The simulation procedures of ground motion accelerograms and tsunami waves are explained in Sects. 3 and 4, respectively. In Sect. 5, the developed computational tool is implemented for the 2011 Tohoku earthquake to demonstrate its performance in comparison with observed ground motion and tsunami data. Finally, concluding remarks on further extensions/applications of the developed tool are mentioned in Sect. 6.

## Analytical framework for coupled earthquake–tsunami simulation

### Methodology

**Fig. 1:**
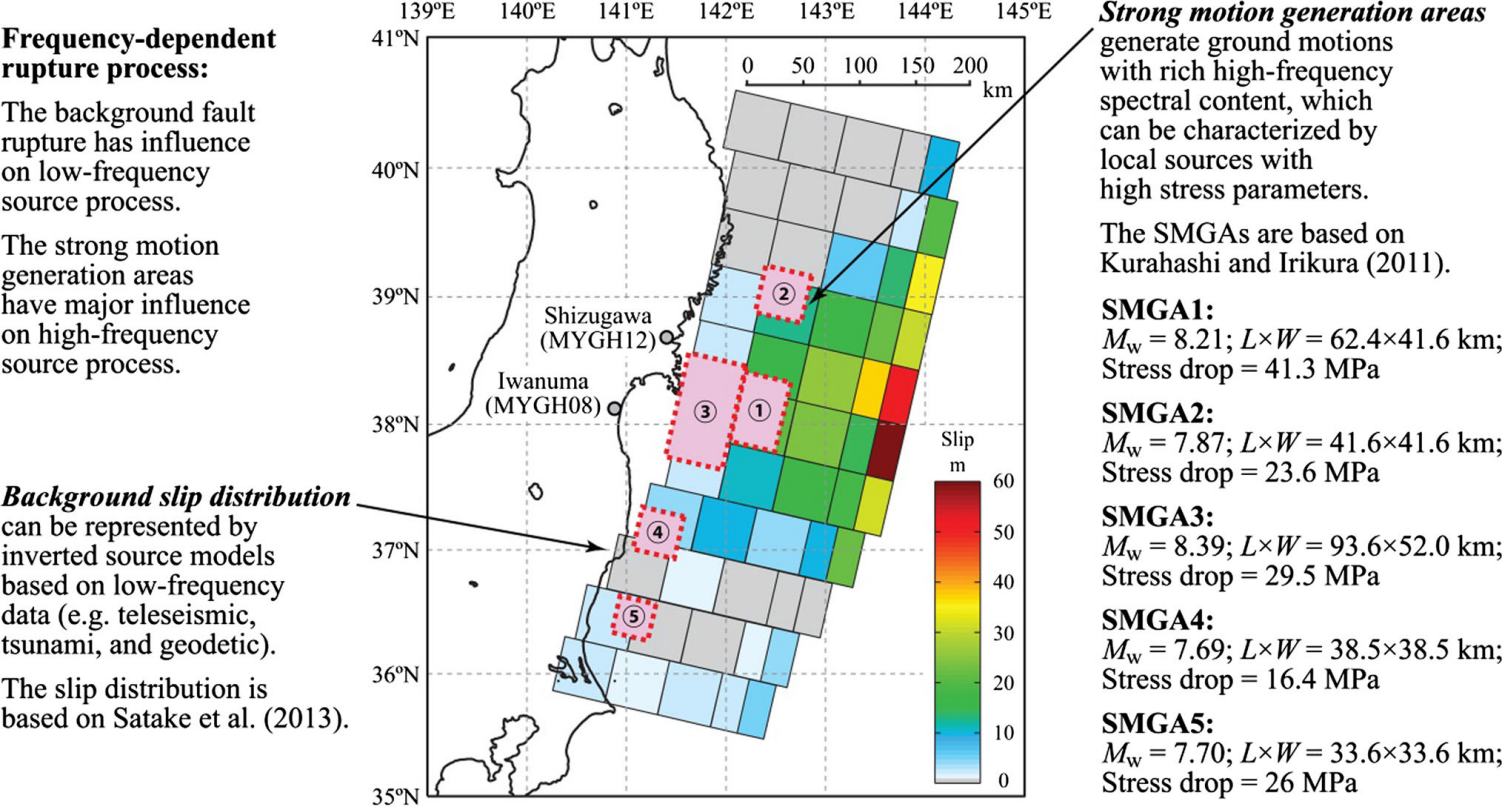
Earthquake rupture process of strong motion and tsunami for the 2011 Tohoku earthquake

A computational framework for concurrently simulating shaking-tsunami sequences is developed by considering the dependency of the two hazard processes on earthquake source characteristics explicitly. The method is based on the state-of-the-art source modeling approaches for mega-thrust subduction earthquakes. The low-frequency process is represented by stochastic source models for the background fault rupture (Goda et al. 2014), whereas the high-frequency process is modeled by SMGA-based source models (Ghofrani et al. 2013; Goda et al. 2015a). The interaction between the background fault rupture and SMGAs is determined based on seismic moment and fault area constraints. A graphical representation of the source modeling is shown in Fig. 1, displaying a slip model by Satake et al. (2013) and a rupture model by Kurahashi and Irikura (2011), as examples, for the background slip distribution and the SMGAs, respectively.

The macroscopic parameters, such as seismic moment, fault rupture area and stress drop parameter, are determined based on scaling relationships and information specific to a given scenario (i.e. 2011 Tohoku earthquake in this study). To account for uncertainties of the background source process, slip distributions that have similar slip statistics and spatial features to an inverted source model are generated using the spectral synthesis method (Mai and Beroza 2002). The analysis procedure of this method is summarized in Sect. 2.2. As inverted source models differ significantly (Goda et al. 2014; Razafindrakoto et al. 2015), multiple reference models are considered to account for epistemic uncertainties related to earthquake source modeling (Ammon et al. 2011; Fujii et al. 2011; Hayes 2011; Iinuma et al. 2011, 2012; Shao et al. 2011; Yamazaki et al. 2011; Gusman et al. 2011; Satake et al. 2013). Generally, the number and locations of SMGAs within a background fault plane can be selected based on the past regional seismicity as well as seismotectonic features. Seismic moment, stress drop parameter and average slip of the SMGAs can be determined using empirical scaling relationships (Somerville et al. 1999; Irikura and Miyake 2011; Central Disaster Management Council 2012; see Sect. 2.3). Because the seismic moment, stress drop parameter and slip are physically related (Stein and Wysession 2003), these parameters need to be chosen consistently. Furthermore, the key SMGA parameters are varied based on statistical distributions. See Sect. 2.4 for the determination of the strong motion parameters.

**Fig. 2:**
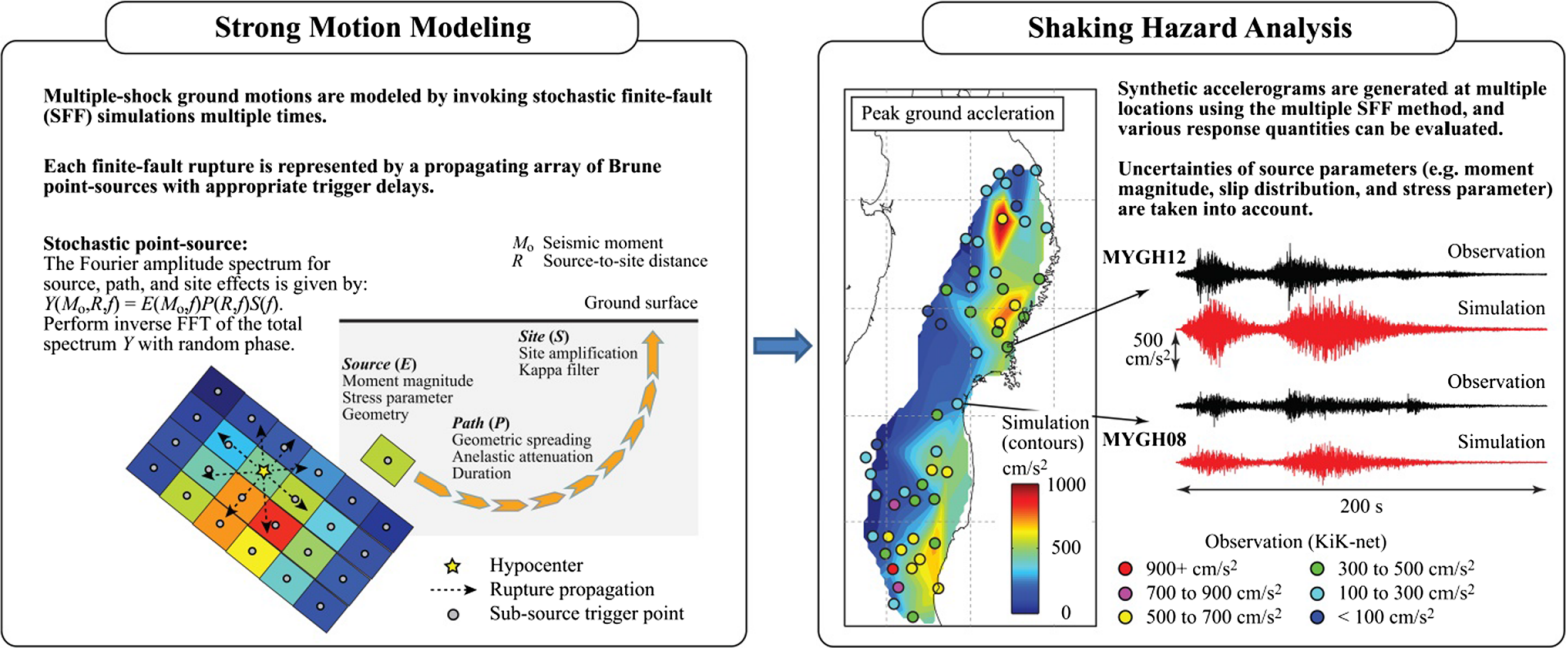
Stochastic finite-fault modeling and seismic hazard analysis based on simulated ground motion accelerograms

**Fig. 3:**
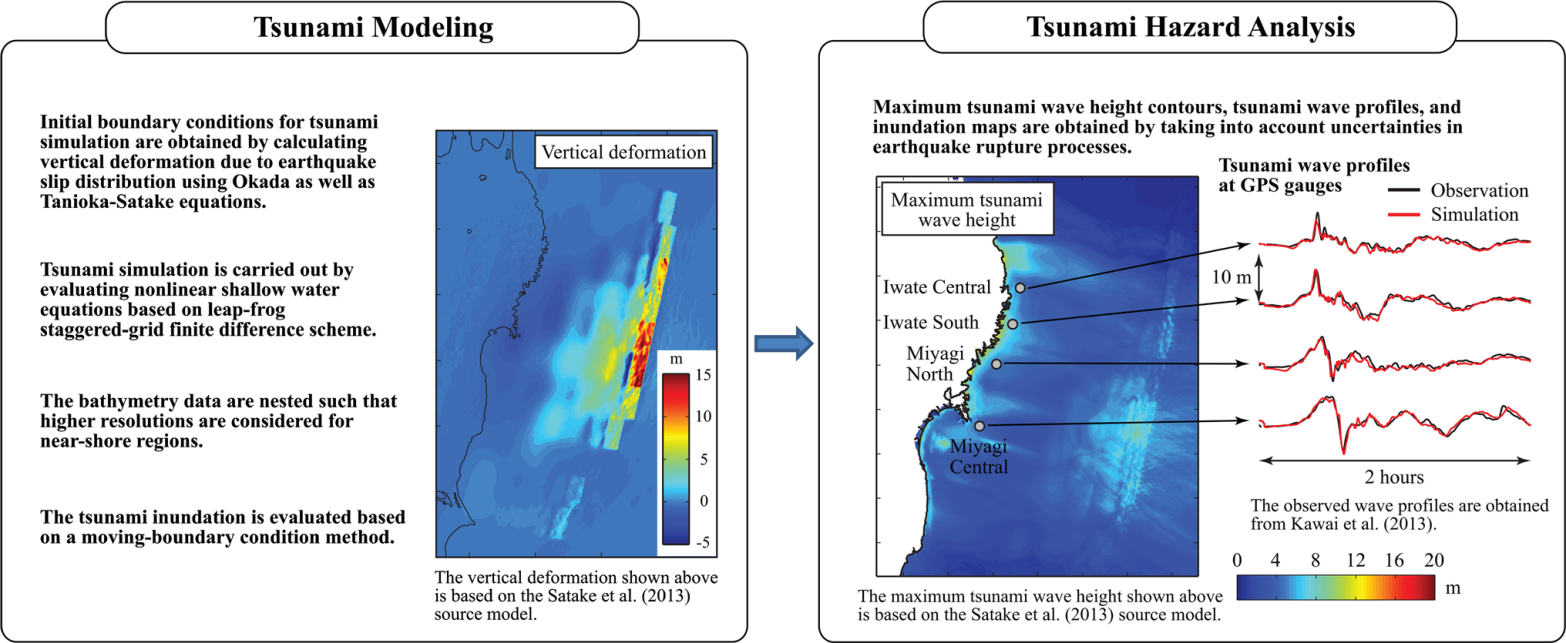
Tsunami modeling and tsunami hazard analysis based on simulated wave time-histories

Using the generated earthquake source models (i.e. background slip distribution and SMGAs), the multiple-event SFF method is implemented to carry out strong motion simulations. Subsequently, synthetic accelerograms are obtained at multiple locations and shaking hazards can be evaluated (e.g. contour maps of ground motion intensity measures). The procedure is illustrated in Fig. 2 and more details of the analysis method are given in Sect. 3. At the same time, using the stochastic source models, initial boundary conditions due to earthquake deformation are computed for tsunami simulation; and tsunami wave propagation and inundation are evaluated by solving the nonlinear shallow water equations. Eventually, tsunami wave time-histories and inundation hazard parameters can be obtained for multiple earthquake scenarios. The procedure is illustrated in Fig. 3 and more details of the analysis method are given in Sect. 4. In short, the developed tool can be used for simulating coupled hazard processes as well as for assessing their sensitivity to variable source characteristics.

### Stochastic source modeling and synthesis

**Fig. 4:**
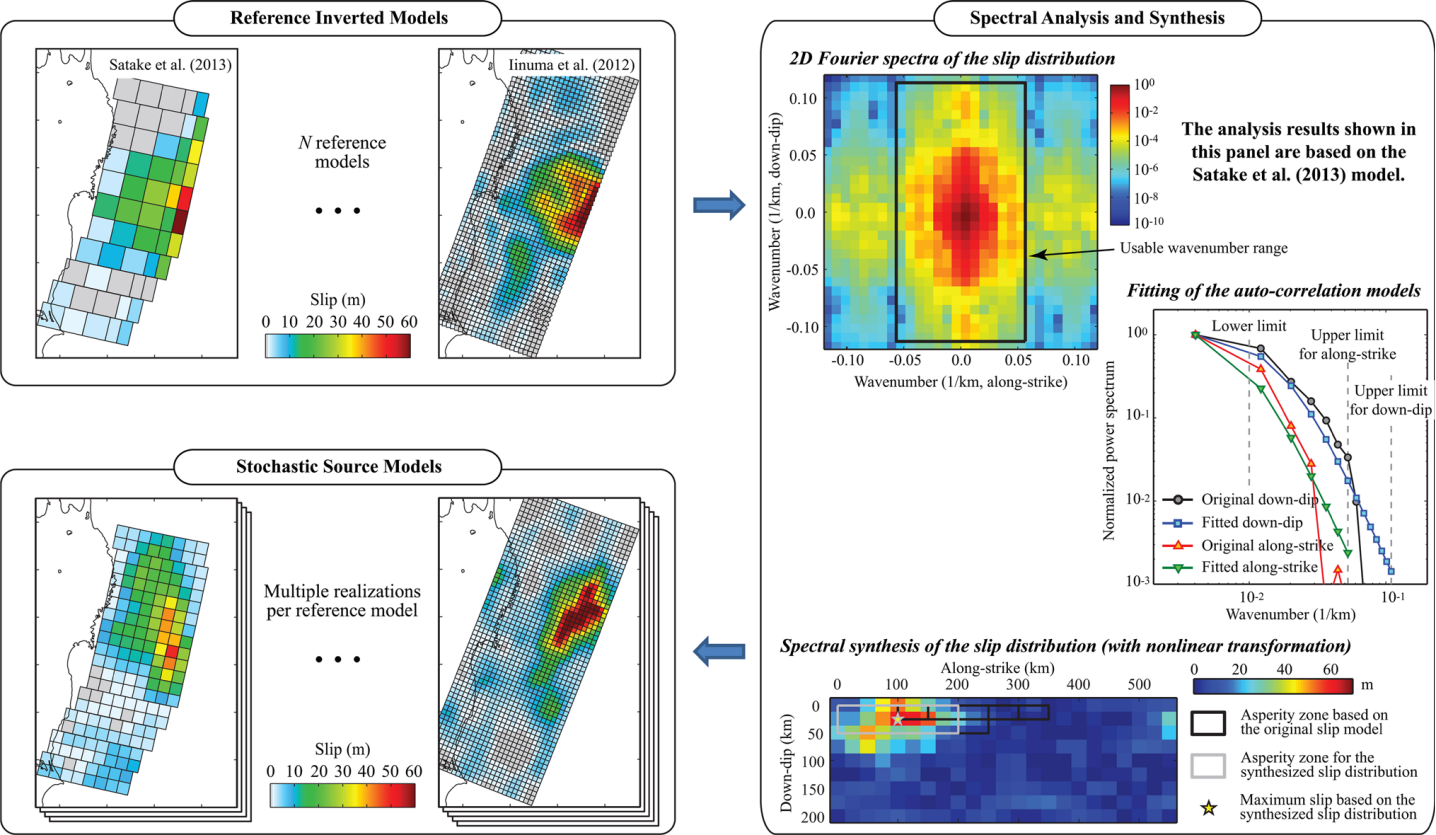
Spectral synthesis of stochastic source models for the background fault rupture

The slip distribution of the background fault rupture is modeled through spectral synthesis of random fields (Mai and Beroza 2002). In this method, spatial characteristics of earthquake slip are expressed in terms of wavenumber spectra of a reference inverted source model. The synthesized slip distributions are used for both strong motion and tsunami simulations. A brief summary of the stochastic method is given below and the analysis procedure is illustrated in Fig. 4; full details of the method can be found in Goda et al. (2014, 2015b).

**Fig. 5:**
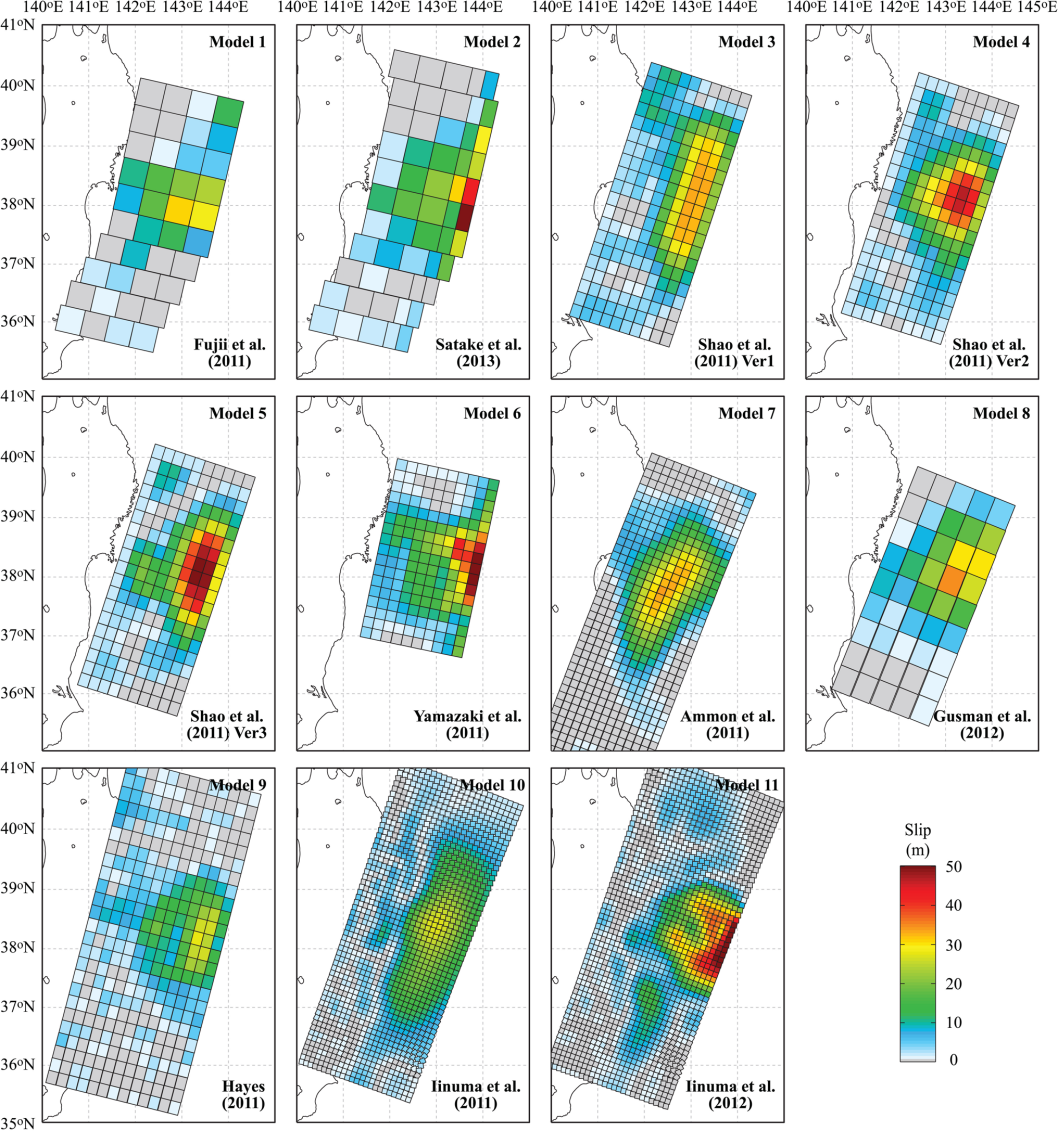
11 reference models for the background rupture

The starting point for the stochastic simulation of earthquake source models is to select a set of suitable inverted models that capture the low-frequency rupture process (see *Reference Inverted Models* panel in Fig. 4). These models are used as a reference to further synthesize stochastic slip distributions that resemble key features of the reference models. For well-recorded earthquakes, numerous inversion models that are derived from different observations and inversion techniques are available and can be adopted as a reference. For instance, Goda et al. (2014) used 11 reference source models for the 2011 Tohoku earthquake, which are shown in Fig. 5, to develop 550 stochastic source models (i.e. 50 scenarios per reference model). The macroscopic features of the source models differ significantly (Table 1); e.g. among the 11 inverted models, the moment magnitude ranges from 8.94 to 9.11 (more than a factor of 2 difference in terms of seismic moment release), the fault length ranges from 340 to 625 km, and the fault width ranges from 200 to 260 km. In addition, the adopted reference models have different geometry and asperity characteristics, such as location, size and amplitude, reflecting the complexity and uncertainty in imaging the 2011 Tohoku rupture process. Adopting multiple background rupture models is advantageous in capturing epistemic uncertainty of the source modeling. In this study, the same set of 550 stochastic source models that were developed by Goda et al. (2014) is considered.

**Table 1:** Summary of 11 reference models

Model ID and reference	Moment magnitude	Length (km)	Width (km)	Strike, dip, rake (°)	Data type
1: Fujii et al. (2011)	8.99	500	200	[193, 14, 81]	Tsunami
2: Satake et al. (2013)	9.02	550	200	[193, 8–16, 81]	Tsunami
3: Shao et al. (2011) [Ver1]	9.10	500	200	[198, 10, *Var* ^a^]	Teleseismic
4: Shao et al. (2011) [Ver2]	9.11	475	200	[198, 10, *Var*]	Teleseismic
5: Shao et al. (2011) [Ver3]	9.11	475	200	[198, 10, *Var*]	Teleseismic
6: Yamazaki et al. (2011)	8.94	340	200	[192, 12, *Var*]	Teleseismic & tsunami
7: Ammon et al. (2011)	8.97	600	210	[202, 12, 85]	Teleseismic & geodetic
8: Gusman et al. (2012)	9.07	450	200	[202, 5-20, *Var*]	Tsunami & geodetic
9: Hayes (2011)	9.06	625	260	[194, 10, *Var*]	Teleseismic
10: Iinuma et al. (2011)	9.00	600	240	[*Var*, *Var*, *Var*]	Geodetic
11: Iinuma et al. (2012)	9.00	620	260	[*Var*, *Var*, *Var*]	Geodetic

^a^ *Var* represents that the parameter is variable

The wavenumber power spectrum of spatial slip distribution is modeled based on a von Kármán auto-correlation function (Mai and Beroza 2002):

1 $$P(k) \propto \frac{{A_{dip} A_{strike} }}{{(1 + k^{2} )^{H + 1} }},$$

where *k* is the wavenumber, *k* = (*A*_dip_^2^ *k*_dip_^2^ + *A*_strike_^2^ *k*_strike_^2^)^0.5^. In the von Kármán model, *A*_dip_ and *A*_strike_ capture the anisotropic spectral features of the slip distribution in down-dip and along-strike directions, respectively, and control the power spectrum in the low wavenumber range (i.e. *k* ≪ 1; long wavelength). *H* determines the slope of the power spectral decay in the high wavenumber range (i.e. short wavelength), and is theoretically constrained to fall between 0 and 1. For each reference source model, *A*_dip_, *A*_strike_ and *H* are estimated by minimizing the differences between the observed down-dip/along-strike spectrum and the theoretical spectrum (see *Spectral Analysis and Synthesis* panel in Fig. 4). Realizations of random-field slip distributions with desirable spectral features are generated using a Fourier integral method (Pardo-Iguzquiza and Chica-Olmo 1993), in which the target amplitude spectrum is defined as in Eq. (1) with estimated values of *A*_dip_, *A*_strike_ and *H*, while the phase spectrum is represented by a random phase matrix (uniform distribution between 0 and 2π). The constructed complex Fourier coefficients are transformed into the spatial domain via two-dimensional inverse fast Fourier transform (FFT). To ensure that the synthesized slip distributions resemble the reference model in terms of location and amplitude of high-slip patches, the asperity dimensions of the synthesized distribution are qualitatively compared with the reference slip distribution. An acceptable slip distribution is required to have its maximum slip patch within the asperity zone of the reference distribution, with its slip concentration located in the rectangular asperity zone greater than the threshold value given by the reference model. The asperity zone of the reference model is determined as a set of sub-faults that have slip values greater than three times the average slip. The slip concentration within the asperity region varies between 0.25 and 0.45 and depends on the reference model (Goda et al. 2014). The synthesized slip distributions that do not meet the criteria are discarded, and the stochastic synthesis is repeated until an acceptable slip distribution is generated. Subsequently, to achieve realistic features of the slip distribution having positive skewness (Goda et al. 2014), the synthesized slip distribution is converted via power transformation. The transformed slip distribution is further adjusted to achieve the target mean slip (to match with the total seismic moment of the reference source model) and to avoid very large slip values exceeding the observed maximum slip of the reference model. By repeating the stochastic synthesis procedure for all reference source models, numerous stochastic source models that capture different source features can be generated (see *Stochastic Source Models* panel in Fig. 4).

### Current approaches for source characterization for strong motion simulation

There are several procedures for defining strong motion source parameters of the background fault rupture and SMGAs for shallow crustal earthquakes (e.g. Irikura and Miyake 2011; Morikawa et al. 2011). In light of key findings from the 2011 Tohoku earthquake, the Central Disaster Management Council (2012) adapted these methods to determine the source parameters (i.e. rupture area, seismic moment, stress drop parameter and average slip) for mega-thrust subduction earthquakes that are expected in the Nankai–Tonankai region of Japan. The implemented procedure serves as useful guidance in selecting ranges of the source parameters for the background rupture and SMGAs. It is noted that the strong motion simulation method that is implemented in this study (Sect. 3) is not directly based on the approach by the Central Disaster Management Council (2012), which is summarized in the subsequent paragraph.

In the source characterization by the Central Disaster Management Council (2012), two scaling relationships were mainly considered. The first one relates seismic moment (*M*_o_) to fault area (*S*) and stress drop (Δσ) by considering multiple circular cracks (Boatwright 1988):

2 $$M_{o} = \frac{16}{{7\pi^{1.5} }}\Delta \sigma S^{1.5} ,$$

whereas the second one relates average slip (*D*) to seismic moment and fault area:

3 $$D = M_{o} /\mu S,$$

where *μ* is the rock rigidity. In applying these relationships to the background fault rupture and SMGAs, it was considered that the fault areas for the background rupture and SMGAs are 90 and 10% of the entire rupture area, respectively, and that the average slip for the SMGAs is twice as large as the average slip of the entire fault rupture. These assumptions were based on the empirical findings from past major earthquakes (Somerville et al. 1999; Central Disaster Management Council 2012). In the development process of the source model, first, the source area and seismic moment for the entire fault rupture were selected (based on seismotectonic knowledge of the region), and the rupture areas and seismic moments for the background fault plane and the SMGAs were determined using: *S* = *S*_background_ + *S*_SMGA_ and *M*_o_ = *M*_o,background_ + *M*_o,SMGA_. The relationships shown in Eqs. (2) and (3) were then applied to the background fault rupture and SMGAs to obtain the source parameters. The stress drop parameters for the SMGAs were significantly greater than that for the background fault plane (typically, one order difference).

It is important to point out that the preceding method only reflects the current understanding of the complex source rupture process of mega-thrust subduction earthquakes. Moreover, source characterizations for strong motion and tsunami differ among different working groups of the Central Disaster Management Council. This situation essentially reflects the fact that there is no unified framework for developing source models of mega-thrust subduction earthquakes that fully explain frequency-dependent rupture processes. Modeling interdependency of the source parameters for the background fault rupture and SMGAs, beyond empirical relationships, is an open research field that needs further improvements in the future.

### Statistical information of source parameters for strong motion simulation

The uncertain characteristics of the background rupture can be accounted for by using stochastic source models having variable geometry, seismic moment and slip distribution (Sect. 2.2). In the SFF-based strong motion simulation (see Sect. 3), the stress drop parameter for the background fault rupture needs to be specified. The possible range of the stress drop parameter for the background rupture can be obtained by calibrating the SFF model using the observed ground motion data. Ghofrani et al. (2013) estimated as Δσ = 3.5 MPa using near-source ground motion data of the 2011 Tohoku earthquake. It can also be constrained based on the statistical information of this parameter evaluated in various past studies. For instance, investigations of the stress drop parameter for global and major subduction earthquakes by Allmann and Shearer (2009) and Ye et al. (2016) suggested Δσ = 4.0 MPa. Furthermore, the statistical analysis of the stress drop parameter conducted by Allmann and Shearer (2009) showed that the stress drop parameter can be modeled by the lognormal distribution with the coefficient of variation (CoV) between 0.3 and 0.4. Such statistical information is particularly valuable when the uncertainty of the key source parameters is taken into account in stochastic strong motion simulation.

Moreover, the stress drop parameter of SMGAs has a major influence on the simulated ground motions at high frequencies (Stein and Wysession 2003; Ghofrani et al. 2013). Thus it is important to take into account the uncertainty associated with this parameter in strong motion simulation. A review of six SMGA-based inversion studies for the 2011 Tohoku earthquake, carried out by the Central Disaster Management Council (2012), indicated that the stress drop parameter for SMGAs takes values ranging from 10 to 50 MPa. The mean and standard deviation of the stress drop parameter for the SMGAs were estimated as 24 and 8.5 MPa, respectively (i.e. CoV = 0.35). In addition, the area ratios between the SMGAs and background fault plane were in a range between 0.05 and 0.13 with the average value of 0.075. The seismic moment ratios between the SMGAs and background fault plane for the six studies were more variable than the area ratios. Their range was from 0.03 to 0.28, and the average seismic moment ratio was 0.12. The source models for the Nankai–Tonankai earthquake, developed by the Central Disaster Management Council (2012), were determined based on the indicated ranges of the rupture area ratios and the seismic moment ratios.

Other critical source parameters are the number and the locations of SMGAs within the background fault plane, because they essentially determine multiple-shock features of an earthquake. Currently, there is no method for determining these parameters that is universally applicable to all situations. In the source inversion studies (e.g. Kurahashi and Iikura 2013), the number and locations of SMGAs were determined by identifying major wave packets in the recorded ground motions using the back-propagation method. For instance, rupture models by Asano and Iwata (2012) and Kurahashi and Iikura (2013) had four and five SMGAs, respectively. For future events, seismological knowledge in the region of interest can also be utilized to select the appropriate number of SMGAs and their locations.

## Multiple-event stochastic finite-fault simulation of strong motion

### Stochastic finite-fault modeling

The SFF method approximates ground motions as a propagating array of Brune point sub-sources, each of which can be modeled as a stochastic point source. See *Strong Motion* panel in Fig. 2. The amplitude spectrum of the total ground motion at a site of interest is defined by multiplying source, path and site spectra in the frequency domain as (Boore 2003):

4 $$Y(M_{o} ,R,f) = E(M_{o} ,f)P(R,f)S(f),$$

where *E*(*M*_o_,*f*) is the source spectrum that is characterized by the seismic moment *M*_o_ and other geophysical parameters (e.g. radiation pattern), *P*(*R*,*f*) is the path spectrum that captures the geometrical spreading and anelastic attenuation of seismic waves as a function of source-to-site distance *R*, *S*(*f*) is the site spectrum that is characterized by the near-surface soil properties and the high-frequency cut-off filter, and *f* is the frequency.

In the stochastic point-source method (Boore 2003), white noises are first generated in the time domain and then transformed into the frequency domain via FFT. The amplitude spectrum of the white noises is then modified by the Fourier spectrum of the ground motion (Eq. 4). Subsequently, inverse FFT is applied to the filtered white noises to obtain a simulated acceleration time-history. The low-frequency spectral level is determined by the moment magnitude of an entire fault plane, whereas the high-frequency spectral content is controlled by the stress parameter of sub-sources. Each sub-source is activated once, with an appropriate delay time based on rupture propagation from a hypocenter to the sub-source. The acceleration time-history from the entire fault rupture, *A*(*t*), is given by:

5 $$A(t) = \sum\limits_{i = 1}^{n} {\sum\limits_{j = 1}^{m} {H_{ij} \times A_{ij} (t - \Delta t_{ij} )} } ,$$

where *n* and *m* are the number of sub-sources along the length and width of the fault plane, respectively, *H*_ij_ is a normalization factor for the *ij*-th sub-source that aims to conserve energy (Motazedian and Atkinson 2005), *A*_ij_(*t*), is the signal of the *ij*-th point-source activation, and Δ*t*_ij_ is the relative time delay for the radiated seismic wave from the *ij*-th sub-source to reach the observation point. The EXSIM code developed by Boore (2009) synthesizes acceleration time-history data using a dynamic corner frequency approach, in which the corner frequency of newly activated sub-sources shifts to lower frequencies as the rupture area grows. The dynamic corner frequency approach makes the spectral shape and spectral level of the resultant accelerograms independent of sub-source size (Motazedian and Atkinson 2005).

### Strong motion simulation of the 2011 Tohoku earthquake

To model the observed multiple-shock features of the 2011 Tohoku earthquake, Ghofrani et al. (2013) extended the SFF method to consider multiple events (i.e. ruptures). Specifically, the multiple-event SFF method triggers stochastic simulations multiple times for the background fault plane and SMGAs with delays. Its key feature is that the complexity and heterogeneity of source characteristics are modeled explicitly by assigning different model parameters to individual rupture segments radiating intense high-frequency energy. The multiple-event SFF model for the 2011 Tohoku mainshock was calibrated using real ground motions recorded at the KiK-net stations (Ghofrani et al. 2013; Goda et al. 2015a). The SMGA parameters (i.e. size, location, moment magnitude, stress drop parameter and rupture delay time) were based on Kurahashi and Irikura (2011) (see Fig. 1). Availability of both borehole and ground surface recordings at the KiK-net stations facilitated the effective separation of local site effects from the source and path effects, resulting in more accurate ground motion modeling. In the calibration, the stress drop parameter for the background fault plane and the corner frequencies of the matching filters of the SMGAs were treated as free parameters. The calibration was carried out primarily by comparing real versus simulated ground motions in terms of 5%-damped elastic response spectra. The stress drop parameter for the background fault plane was estimated as 3.5 MPa (which is consistent with the global average of this parameter; Allmann and Shearer 2009; Ye et al. 2016), whereas the corner frequencies of the matching filters were in a range between 0.3 and 0.6 Hz (Ghofrani et al. 2013).

The strong motion simulation based on the multiple-event SFF method starts with fault rupture of the background plane. Subsequently, SMGAs are triggered with time delays to account for realistic rupture propagation within the fault plane. Synthesized time-history data from the SMGA sources are processed using a causal low-cut matching filters, and then filtered data are summed in the time domain as follows:

6 $$A_{total} (t) = A_{b} (t) + \sum\limits_{k = 1}^{M} {F_{k} * A_{k} (t - \Delta T_{k} )} ,$$

where *A*_b_(*t*) and *A*_k_(*t*) (*k* = 1 to *M*; *M* is the number of SMGAs) are the simulated acceleration time-histories of the background plane and SMGAs, and are synthesized as in Eq. (5), Δ*T*_*k*_ is the time delay between the nucleation point of the *k*-th SMGA and the hypocentre of the background plane, and *F*_k_ is the low-cut filter with the specified corner frequency. It is noted that the simulated time-history from the background plane is not filtered when the summation is carried out. Thus low-frequency components of the synthesized ground motions are mainly contributed by the background plane, whereas SMGAs have a significant influence on high-frequency components. The residual plots between the calibrated model and observed recordings at 48 KiK-net stations, presented in Goda et al. (2015a), indicated that the median curves fluctuate around zero (i.e. on average, unbiased) and variability of the residuals at different locations is about 0.2–0.3 log_10_ units, depending on the vibration period (note: 0.3 log_10_ unit corresponds to a difference by a factor of 2). Overall, there was no particular trend of the misfits in specific period ranges. Moreover, Goda et al. (2015a) carried out statistical validation analyses of the calibrated model based on peak nonlinear responses of inelastic systems with different fundamental vibration periods and strengths. The multiple-event SFF model calibrated for the Tohoku mainshock was capable of estimating inelastic structural responses without significant bias.

## Tsunami wave modeling

### Input data for tsunami simulation

For tsunami simulation, a complete dataset of bathymetry/elevation, coastal/riverside structures (e.g. breakwater and levees), and surface roughness, obtained from the Miyagi prefectural government, is employed. The data are provided in the form of nested grids (1350–450–150–50–10-m), covering the geographical regions of Tohoku. The ocean-floor topography data are based on the 1:50,000 bathymetric charts and JTOPO30 database developed by the Japan Hydrographic Association and based on the nautical charts developed by the Japan Coastal Guard. The raw data are gridded using a triangulated irregular network. The land elevation data are based on a 5-m grid digital elevation model (DEM) developed by the Geospatial Information Authority of Japan. The raw data are from airborne laser surveys and aerial photographic surveys. These data have measurement errors of less than 1.0 m horizontally and of 0.3–0.7 m vertically (as standard deviation). All bathymetry, elevation, and structural height data are defined with respect to Tokyo Peil, which is the standard mean sea level in Japan. The tidal fluctuation is not taken into account in this study.

The elevation data of the coastal/riverside structures are primarily provided by municipalities. In the coastal/riverside dataset, structures having dimensions less than 10 m only are represented, noting that those having dimensions greater than 10 m are included in the DEM. In the simulation, the coastal/riverside structures are represented by a vertical wall at one or two sides of the computational cells. To evaluate the volume of water that overpasses these walls, Honma’s overflowing formulae are employed (Japan Society of Civil Engineers 2002). The bottom friction is evaluated using Manning’s formula. The Manning’s coefficients are assigned to computational cells based on national land use data in Japan (Japan Society of Civil Engineers 2002): 0.02 m^−1/3^ s for agricultural land, 0.025 m^−1/3^ s for ocean/water, 0.03 m^−1/3^ s for forest vegetation, 0.04 m^−1/3^ s for low density residential areas, 0.06 m^−1/3^ s for moderate density residential areas, and 0.08 m^−1/3^ s for high density residential areas.

### Tsunami inundation simulation

Tsunami wave propagation modeling is carried out using a well-tested numerical code (Goto et al. 1997) that is capable of computing on-shore tsunami inundation profiles by evaluating nonlinear shallow water equations with run-up. The run-up calculation is based on a moving boundary approach, where the dry/wet condition of a computational cell is determined based on total water depth relative to its elevation. To compute initial water surface elevation for a given earthquake slip model, analytical formulae for elastic dislocation by Okada (1985) together with the equation by Tanioka and Satake (1996) are used. The latter is to take into account the effects of horizontal movements of the steep seafloor on the vertical water dislocation along the Japan Trench. Numerical tsunami calculation is performed by considering the minimum grid size of 50-m. The use of the 50-m grid resolution dataset, rather than the 10-m resolution dataset (i.e. finest resolution dataset provided by the Miyagi prefectural government), is due to the significant computational requirements when the 10-m resolution dataset is employed for the stochastic tsunami simulations. The simulation duration is set to 2 h with an integration time step of 0.5 s. It is noted that the shallow water formulations of propagating tsunami waves based on Goto et al. (1997) cannot model the dispersive tsunami waves, the effects of which can be noticeable for far-field tsunami wave profiles (Løvholt et al. 2012; Kirby et al. 2013).

The left panel of Fig. 3 shows the vertical water dislocation that is calculated based on the Satake et al. (2013) source model. The tsunami simulation results based on the Satake et al. (2013) source model are shown in the right panel of Fig. 3. The simulation produces tsunami wave profiles at GPS buoy gauges from the NOWPHAS system (Kawai et al. 2013; see *Tsunami Hazard Analysis* panel in Fig. 3) as well as maximum tsunami wave height contours. At sub-municipal levels, inundation footprints can be evaluated (Goda et al. 2015b). Furthermore, uncertainties of tsunami inundation and wave heights associated with future scenarios can be assessed by considering stochastic slip distributions (Goda and Song 2016).

## Applications to the 2011 Tohoku earthquake

A demonstration of stochastic coupled simulation of strong motion and tsunami is presented for a case study of the 2011 Tohoku earthquake. Firstly, a set-up of the numerical example, such as the uncertainty treatment of model parameters of the background fault rupture and SMGAs, is explained in Sect. 5.1. In Sect. 5.2, comparison of the observed earthquake–tsunami data with the simulated results is carried out for two locations in Iwanuma and Shizugawa of Miyagi Prefecture. The effects of uncertain model parameters on the simulated strong motion and tsunami are investigated in detail. The aim of the application is to highlight the capability and utility of the developed tool for the coupled earthquake–tsunami simulation in assessing the cascading multi-hazards by taking into account uncertainties. The results will also serve as sensitivity analysis of strong motion-tsunami simulations in comparison with actual observations. In Sect. 5.3, strong motion and tsunami simulation results at multiple sites along the coastline of Miyagi Prefecture are discussed. The aim of this investigation is to examine the joint spatial variation and uncertainty of predicted ground motion and tsunami hazard parameters at coastal locations.

### Calculation cases

Four calculation cases are set up (Table 2). The base case adopts the background fault parameters based on Satake et al. (2013) (i.e. reference model 2 in Fig. 5) and the SMGA parameters based on Ghofrani et al. (2013) and Goda et al. (2015a) (note: the reference model for SMGAs is based on Kurahashi and Irikura (2011) and other parameters have been calibrated against KiK-net observations). For the base case, no parametric uncertainty is taken into account. Figure 1 shows the source model of the base case. It is noted that there is inherent stochasticity in the strong motion simulation as individual point sources in SFF simulations involve white noise generation; 100 realizations of accelerograms (per location) are considered to capture this variability.

**Table 2:** Calculation cases of stochastic coupled simulation of strong motion and tsunami

Case	Background rupture	SMGAs
Base (Case 0)	Same as Satake et al. (2013)	Same as Ghofrani et al. (2013) and Goda et al. (2015a); see also Kurahashi and Irikura (2011)
Case 1	Slip distributions are represented by 11 reference source models and 550 stochastic source models as generated by Goda et al. (2014) Stress drop is modeled by the lognormal distribution^a^	Same as base case
Case 2	Same as base case	Stress drop is modeled by the lognormal distribution^b^ Location of the SMGA fault planes is varied^c^ Magnitude of the SMGAs is varied^d^
Case 3	Same as Case 1	Same as Case 2

^a^Mean = 3.5 MPa and CoV = 0.35 with the lower and upper limits of 1 and 10 MPa

^b^Mean values of the individual SMGAs are the same as those suggested by Kurahashi and Irikura (2011) (see Fig. 1) and CoV = 0.35 with the lower and upper limits of 10 and 70 MPa

^c^The location shift of a SMGA fault plane is calculated by the normal distribution with zero mean and standard deviation of the quarter of the characteristic dimension of the fault plane

^d^The magnitude variation is modeled by the truncated normal distribution with the mean values of the original SMGAs (see Fig. 1) and with the standard deviation of 0.1 magnitude unit. The magnitude variation is truncated at mean ± 2 SDs

Case 1 considers the stochastic background rupture, while the SMGA sources are the same as the base case. For the background rupture, in total, 561 different slip distributions are employed; 11 cases are from the existing inverted source models for the 2011 Tohoku earthquake (Fig. 5), while 550 cases are synthesized from the 11 reference models (50 cases per reference model; Goda et al. 2014). Collectively, the 561 source models represent possible source characteristics of the *M*_w_9-class mega-thrust subduction event in the Tohoku region of Japan. Moreover, the stress drop of the background fault rupture is treated as a random variable, represented by the lognormal distribution with mean = 3.5 MPa and CoV = 0.35, truncated at the lower and upper limits of 1 and 10 MPa, respectively.

Case 2 considers the stochastic SMGA sources, while the background rupture is the same as the base case. The stress drops of the SMGAs are modeled by the lognormal distribution. The mean values are the same as those suggested by Kurahashi and Irikura (2011) (see Fig. 1) and CoV values for the SMGAs are set to 0.35, in accordance with the previous studies (see Sect. 2.4). In addition, the lognormal distribution is truncated at the lower and upper limits of 10 and 70 MPa. The variability of the SMGA locations is represented by the normal random variable with zero mean and standard deviation equal to the quarter of the characteristic dimension of the SMGAs (i.e. square root of width and length of the SMGAs). Note that the rupture trigger times of the SMGAs are not varied. Furthermore, the magnitude variation is modeled by the truncated normal distribution with mean values of the original SMGAs (see Fig. 1) and standard deviation equal to 0.1 magnitude unit. The magnitude variation is truncated at mean ± 2 SDs.

Case 3 considers the stochastic variations of the source characteristics for the background rupture (as in Case 1) and for the SMGAs (as in Case 2). The total number of simulations for Cases 1–3 is 561. The results for the 11 reference cases and the 550 stochastic cases are discussed separately in the following subsections; the reference cases are considered to evaluate the variations of earthquake–tsunami simulations when different inverted source models are used, while the stochastic cases are useful to investigate the sensitivity of earthquake–tsunami simulations by taking into account the stochasticity of source parameters. In all calculation cases, the moment magnitude of the background fault rupture for the strong motion simulation is adjusted for those of the SMGAs; in other words, the background moment magnitude is smaller than the value specified in the original studies. For strong motion simulation, results for Cases 1–3 are different because synthesized accelerograms depend on both background rupture and SMGAs. The synthesized accelerograms corresponds to a horizontal component along random orientation. On the other hand, in the stochastic tsunami simulation, SMGAs are not included, and thus only Case 3 (or 1) is relevant.

### Strong motion and tsunami simulations at Iwanuma and Shizugawa

This section focuses on the comparison of the observed and simulated data for Iwanuma (Sendai coastal plain) and Shizugawa (Sanriku ria coast). Their locations are indicated in Fig. 1 as well as Fig. 7. There are KiK-net strong motion stations, i.e. MYGH08 and MYGH12, in Iwanuma and Shizugawa, respectively, and thus actual accelerograms are available. The average shear-wave velocities in the uppermost 30 m at MYGH08 and MYGH12 are 200 and 750 m^−1^ s, respectively. Because the MYGH08 station is located relatively inland and was not inundated during the 2011 tsunami (Fig. 7), a nearby location along the coastal line is adopted for the tsunami simulation results in Iwanuma. More specifically, Site 3 shown in Fig. 7 is substituted for MYGH08. These two sites are selected because of availability of the data, proximity to earthquake rupture and different features of the topography (coastal plain versus ria coast).

**Fig. 6:**
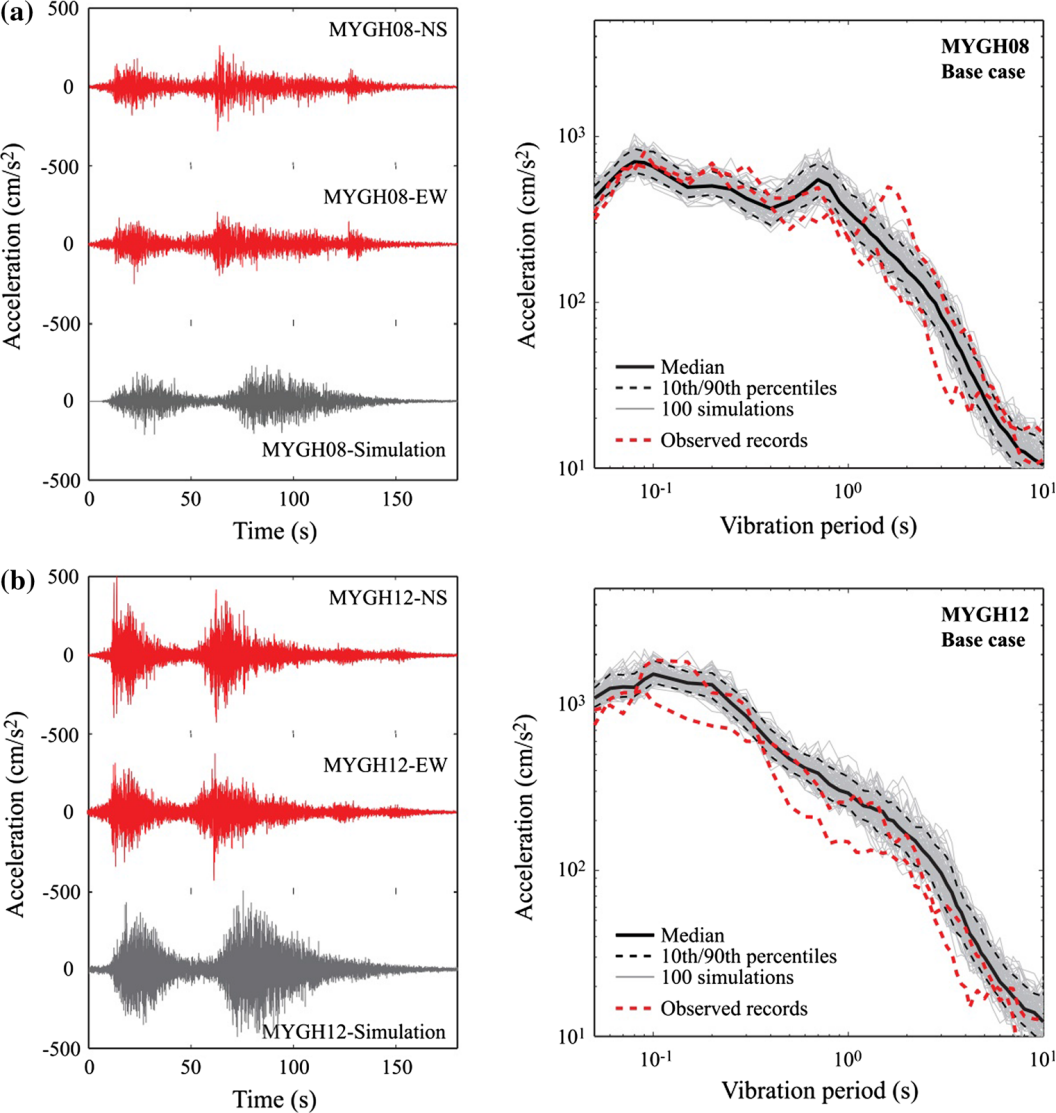
Comparison of observed ground motions and simulated ground motions (base case) in terms of accelerograms and response spectra: **a** MYGH08 and **b** MYGH12

The actual strong motion recordings and simulated accelerograms for the base case are compared in Fig. 6a. The simulated records successfully capture multiple-shock features of the recorded ground motions at MYGH08 and MYGH12. Figure 6b compares 5%-damped response spectra of 100 simulated accelerograms for the base case with those for the observed records at the two stations. The statistics of the simulated response spectra, i.e. median and 10th/90th percentile, are included. For MYGH08, differences between the observed and simulated response spectra are within a factor of 2 over a wide range of vibration periods, achieving on average unbiased estimation. For MYGH12, response spectra of the simulations are greater than those of the observation (on average 20–30% overestimation). Nonetheless, the simulated results capture the key spectral characteristics of the observation well. Such a good match between the observation and the simulations is achieved through the considerations of multiple SMGAs and site-specific site amplification factors at individual KiK-net stations (Ghofrani et al. 2013). Detailed validation results in terms of peak linear and nonlinear structural responses at different KiK-net stations can be found in Goda et al. (2015a). The validation based on nonlinear structural responses ensures that the simulated time-histories from the multiple-event SFF method can be substituted for real strong motion records in nonlinear dynamic analysis and therefore are useful for evaluating the seismic performance of structures.

**Fig. 7:**
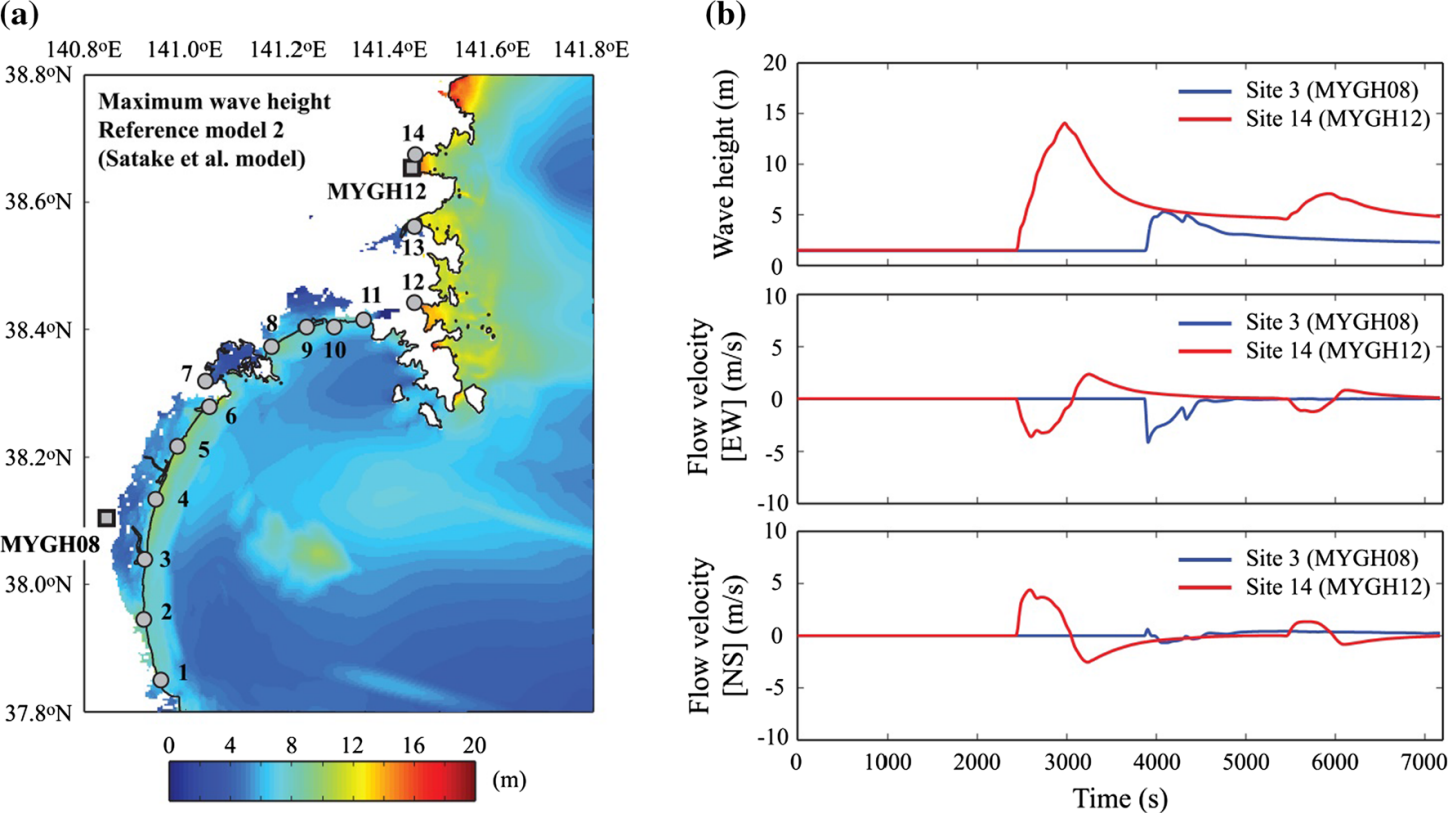
**a** Maximum wave height based on the reference model 2 (Satake et al. 2013), see the locations of MYGH08 and MYGH12 as well as 14 sites along the Miyagi coast, and **b** simulated tsunami waves (wave height and flow velocities in EW and NS directions) at Site 3 and Site 14

Figure 7a shows the maximum wave height map based on the reference model 2 (Satake et al. 2013). In the figure, locations of MYGH08 and MYGH12 as well as 14 sites along the Miyagi coast are also included. To inspect temporal features of the tsunami waves, wave height as well as flow velocity profiles at Sites 3 and 14 are shown in Fig. 7b. The wave height is defined with respect to the mean sea level (i.e. Tokyo Peil); non-zero heights shown in the top panel of Fig. 7b correspond to the land elevations at Sites 3 and 14 (i.e. 1.45 and 1.51 m, respectively). The positive flow velocity corresponds to East-ward and North-ward propagating waves, whereas the negative flow velocity corresponds to West-ward and South-ward propagating waves. The same definition and sign convention for wave height and flow velocity are adopted throughout this study. Because the topographical characteristics of the Sendai plain are similar, the substitution of Site 3 to MYGH08 to show the tsunami waves arriving at shoreline is considered to be adequate. From the temporal characteristics of tsunami waves, information, such as arrival time of the major tsunami waves, can be obtained. It is noteworthy that although tsunami wave observations are not available at Sites 3 and 14 during the 2011 Tohoku tsunami, the maximum inundation data are available from the Tohoku Tsunami Joint Survey group (Mori et al. 2011). The comparisons of the observed and simulated tsunami wave heights are favorable near Sites 3 and 14; see Goda et al. (2015b) for further validation results.

**Fig. 8:**
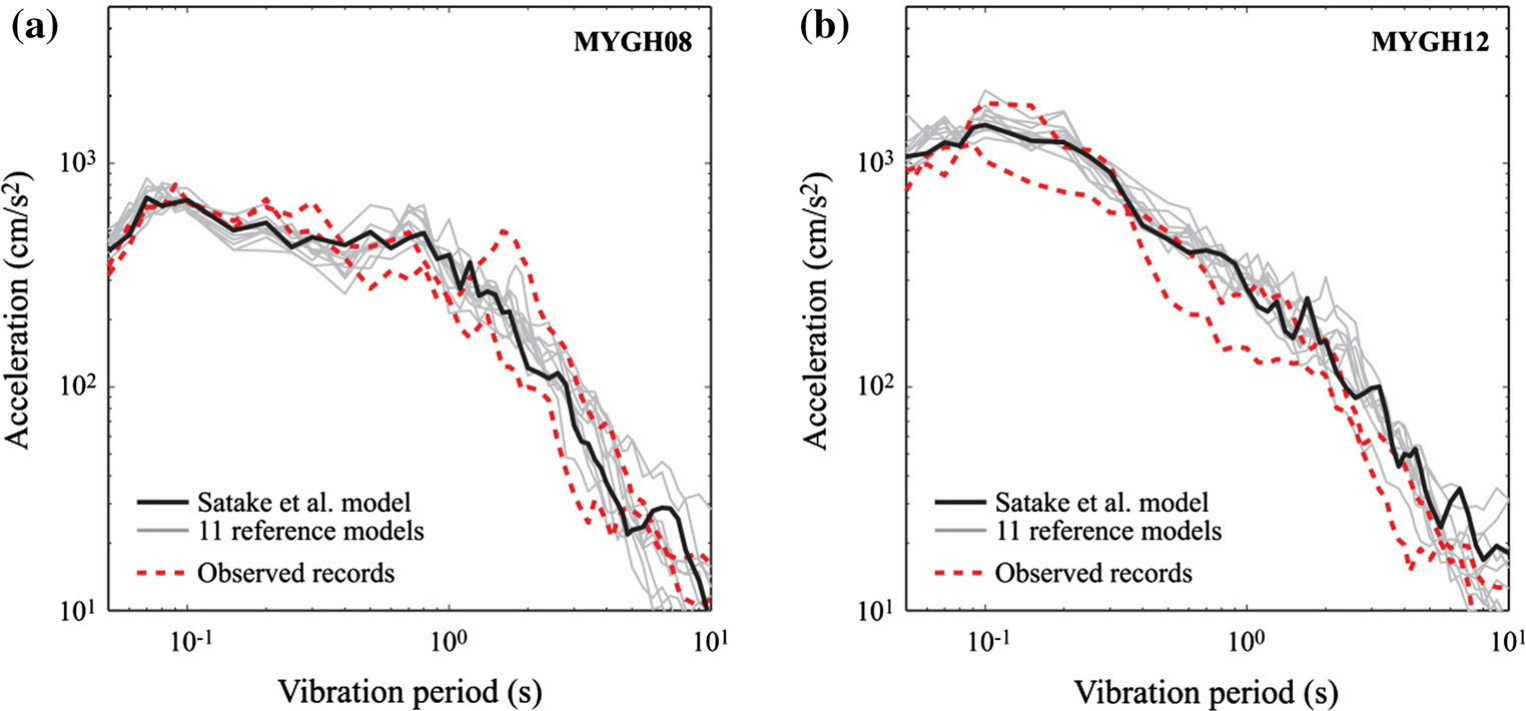
Comparison of response spectra of observed and simulated ground motions (11 reference cases): **a** MYGH08 and **b** MYGH12

Next, the effects of considering different reference models on the simulated strong motion and tsunami are investigated. Figure 8 compares response spectra of observed and simulated ground motions based on the 11 reference models for MYGH08 and MYGH12. The variation of the response spectra in the short vibration period range is due to white noises in SFF simulations. On the other hand, the response spectra in the long vibration period range are affected by the moment magnitude and slip distribution of the inverted source models, resulting in greater variability of the strong motion characteristics.

**Fig. 9:**
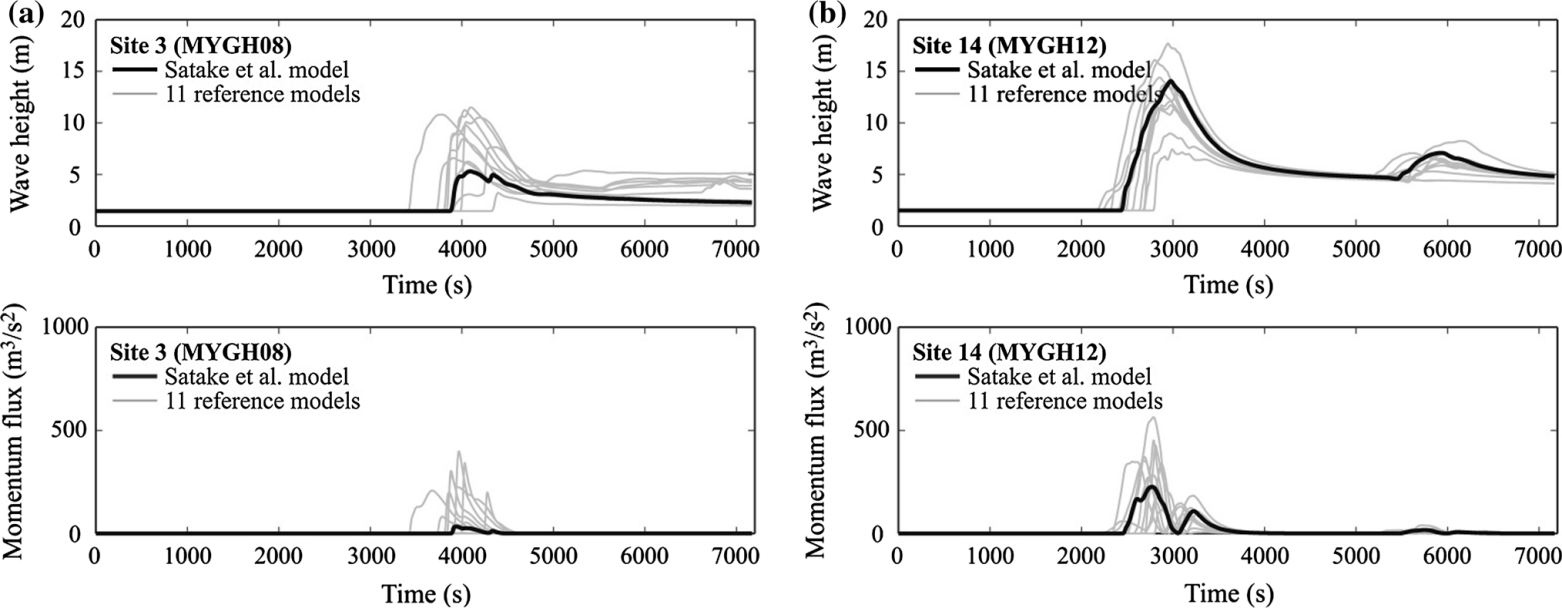
Simulated tsunami wave height profiles and momentum flux profiles (11 reference cases): **a** Site 3 and **b** Site 14

Figure 9 presents the comparison of tsunami waves in terms of wave height profiles and momentum flux profiles for Sites 3 and 14 by considering the 11 reference models. The momentum flux is evaluated by multiplying tsunami inundation depth (i.e. height is adjusted for the elevation) by the squared flow velocity, and is a useful tsunami hazard parameter for tsunami resistance design as it is related to the hydrodynamic forces (Federal Emergency Management Agency 2008). The results shown in Fig. 9 indicate that variability of both tsunami wave height and momentum flux due to the varied source models is large; the peak values of the tsunami hazard parameters can differ by a factor of 2 or more, depending on the source models. It highlights the importance of spatial slip distributions to tsunami simulation results (Goda et al. 2014, 2015b).

**Fig. 10:**
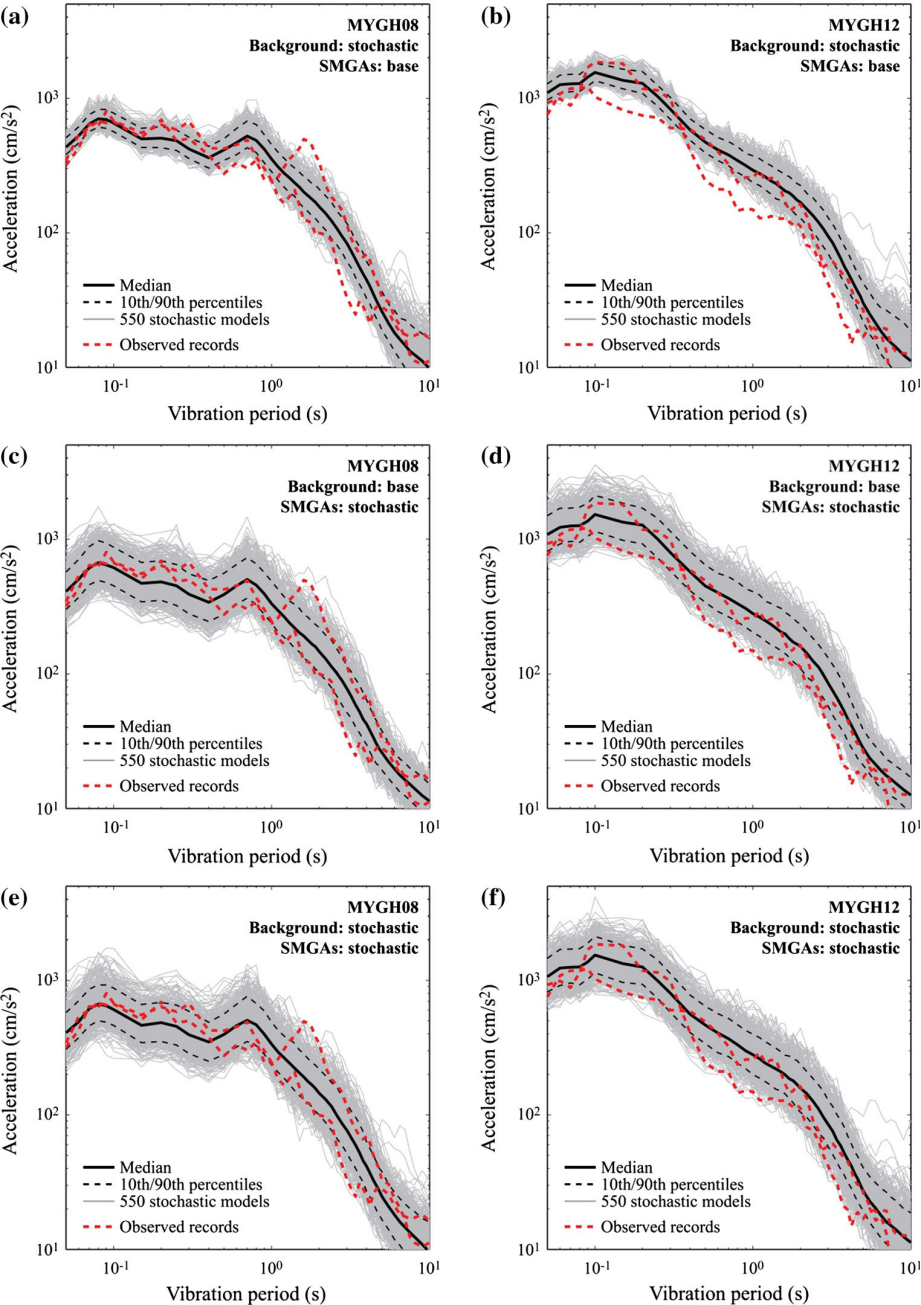
Comparison of response spectra of observed ground motions and simulated ground motions: **a** Case 1-MYGH08, **b** Case 1-MYGH12, **c** Case 2-MYGH08, **d** Case 2-MYGH12, **e** Case 3-MYGH08, and **f** Case 3-MYGH12

To further investigate the effects of incorporating uncertainties of the source parameters on the simulated strong motion and tsunami, sensitivity analysis of simulated ground motions is carried out. The results are presented in Fig. 10. The response spectra of the simulated accelerograms for Cases 1–3 are compared with the observed ground motions at MYGH08 and MYGH12. In the figure, the statistics of the simulated response spectra are also included. In Fig. 10a and b, the background rupture characteristics are varied (Case 1). The results exhibit significant variability in the long vibration period range (particularly, periods longer than 5 s). The increase in the variability of the long-period ground motion components can be seen from the wider interval between the 10th and 90th percentiles of the response spectra, in comparison with the results for the base case shown in Fig. 8. Hence, it is important to capture this uncertainty for the assessment of long-period structures (e.g. high-rise buildings and long-span bridges) subjected to mega-thrust subduction earthquakes. On the other hand, the results for Case 2 (Fig. 10c, d) indicate that greater variation of the response spectra in the short vibration period range is caused by the uncertainties of the SMGA parameters, in comparison with the results for Case 1. Discrepancies can be up to a factor of 3 due to the SMGA characteristics, highlighting the sensitivity of strong motion simulations to fault rupture with large slip velocity. The results for Case 3 (Fig. 10e, f), which accounts for the uncertainties in the background rupture and SMGAs, demonstrate that variability of the strong motion accelerograms can be large, in comparison with the base case (Fig. 8). The fluctuations of the simulated response spectra contain the observed results over almost entire vibration period range. The information on the quantified uncertainty is valuable for making future predictions of the strong motion accelerograms and their spectral characteristics. Overall, the results shown in Fig. 10 help understand the influential model components on different characteristics of strong motions and appreciate the inherent impact of the underlying uncertainties associated with the predictions.

**Fig. 11:**
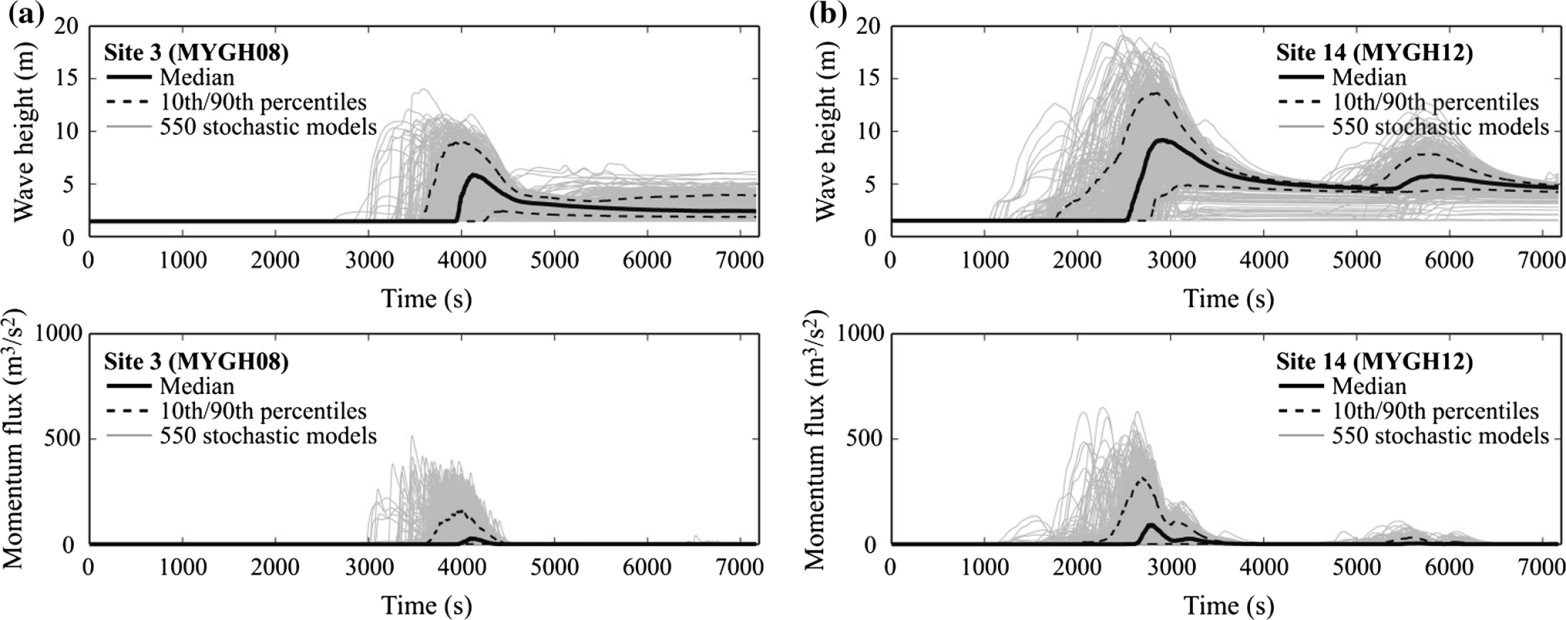
Simulated tsunami wave height profiles and momentum flux profiles (Case 3): **a** Site 3 and **b** Site 14

Figure 11 shows simulated tsunami wave height profiles and momentum flux profiles for Sites 3 and 14 by considering the 550 stochastic source models for the background rupture. The results essentially corroborate the observations made with regard to Fig. 9 by considering the 11 reference models. The variability of the tsunami wave height profiles as well as momentum flux profiles due to different rupture characteristics is significant. This results in a wider range of tsunami scenarios that need to be considered for the design and assessment of tsunami-proof evacuation buildings.

It is important to emphasize that although the results for strong motion simulation and tsunami simulation are presented separately in this section (i.e. in terms of accelerogram or response spectrum and tsunami wave height or momentum flux), these hazard parameters can be converted to forces acting on a building (Petrone et al. 2016), and can be presented as a load sequence. This will facilitate the assessment of the structural performance under cascading earthquake–tsunami loading. This will be a future research topic to which the developed stochastic coupled simulation of strong motion and tsunami can contribute significantly.

### Strong motion accelerograms and tsunami waves along the Miyagi coast

In this section, the coupled strong motion-tsunami simulation is carried out at 14 sites along the Miyagi coast (Fig. 7) to investigate the spatial dependency of ground motion and tsunami hazard parameters. No actual ground motion recordings are available at these locations and thus generic site amplification factors (Ghofrani et al. 2013) are applied in strong motion simulation. The site amplification factors for these sites are specified as average shear-wave velocity in the uppermost 30 m of 360 m^−1^ s.

**Fig. 12:**
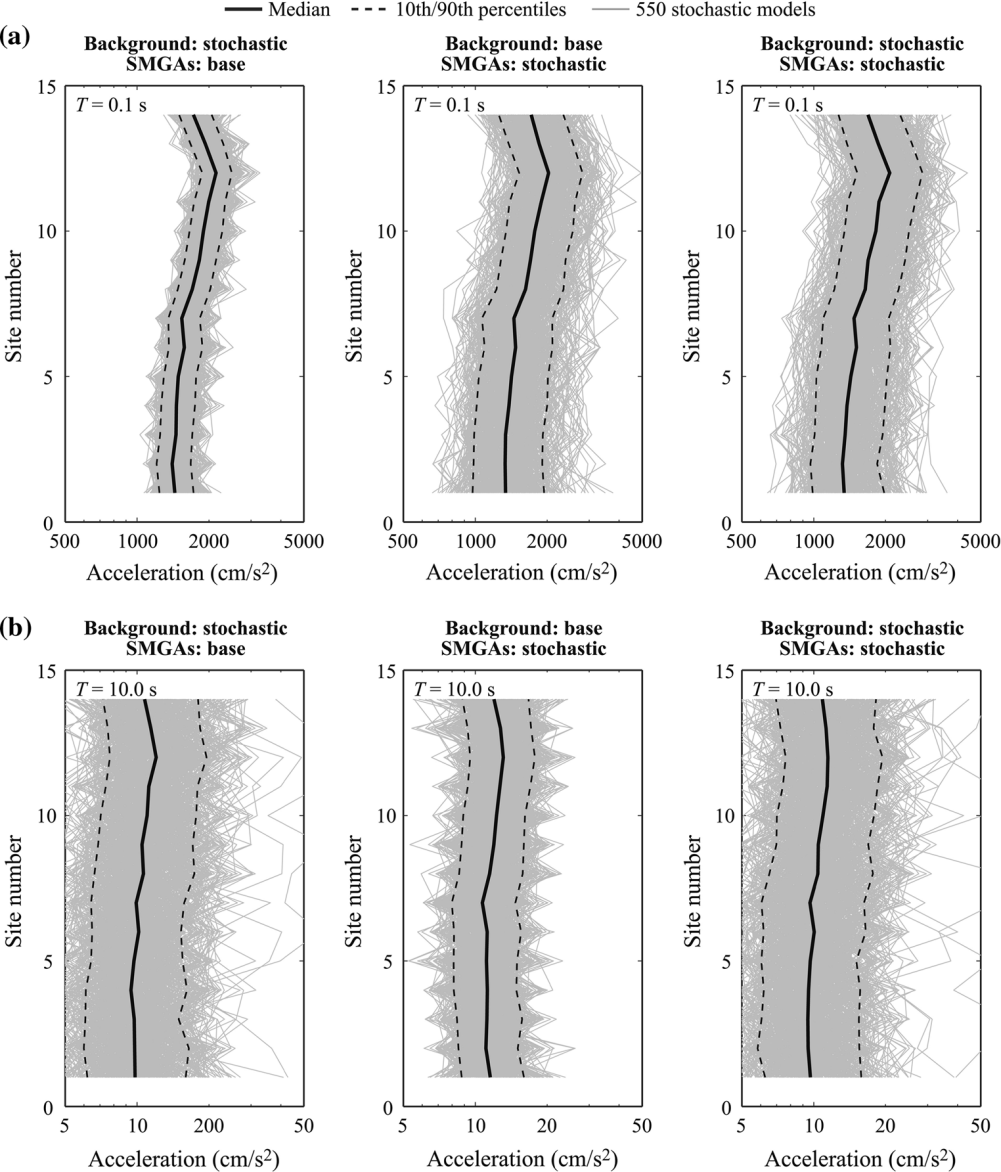
Simulated response spectra at Sites 1–14 (Cases 1–3): **a** *T* = 0.1 s and **b** *T* = 10.0 s

Figure 12 shows simulated response spectra at vibration periods (*T*) of 0.1 and 10.0 s (i.e. short vibration period and long vibration period) by considering Cases 1–3. For *T* = 0.1 s (Fig. 12a), gradual increase of the response spectra from South to North can be observed; this is because northern sites are closer to SMGA3, which is intense (see Fig. 1). When the stochastic SMGA sources are considered (Cases 2 and 3), variability of the strong motion simulations increases significantly. For a given case, the variability of the response spectra is similar at different sites. For *T* = 10.0 s (Fig. 12b), it can be observed that the background rupture has a major influence on variability of the long-period response spectra. The results at intermediate vibration periods (not shown for brevity) are affected by both SMGAs and background rupture. At the regional level, spatial variability of ground motions due to source characteristics is important.

**Fig. 13:**
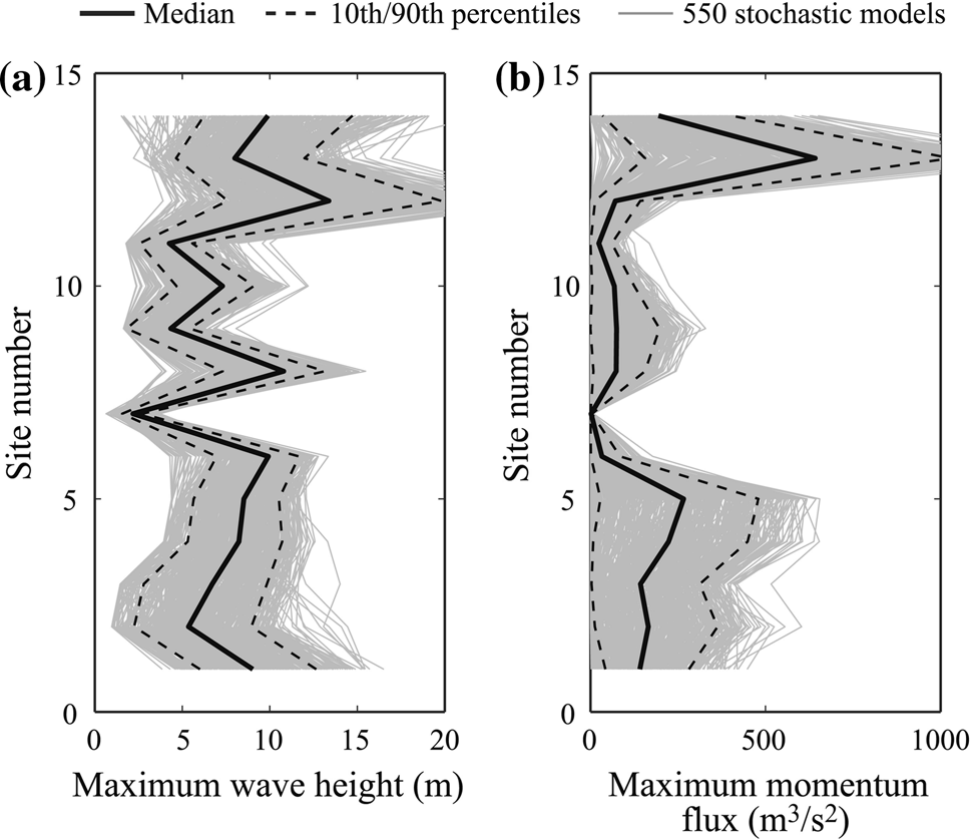
**a** Simulated maximum wave height at Sites 1–14 (Case 3) and **b** simulated momentum flux at Sites 1–14 (Case 3)

To examine the spatial variability of the tsunami hazard parameters, simulated maximum tsunami wave height and momentum flux at Sites 1–14 are plotted in Fig. 13 by taking into account stochastic source models (Case 3). The results indicate that there is remarkable spatial dependency of the tsunami hazard parameters. For the maximum tsunami wave height (Fig. 13a), Sites 1–6 (Soma–Sendai) that are located in the Sendai plain have similar tsunami wave height characteristics. Note that Site 7 is located inside Matsushima Bay; because numerous islands in Matsushima Bay serve as a natural buffer, tsunami waves inside the bay are significantly smaller than those outside. At Sites 8–11 (Higashimatsushima–Ishinomaki), moderate tsunami waves are expected, whereas at Sites 12 –14 (Onagawa–Shizugawa), which are in the ria coast, very large tsunamis are expected. From Fig. 13b, it can be observed that the spatial variability of the maximum momentum flux is more significant than that of the maximum tsunami wave height. This is because the momentum flux includes the effects of flow velocity that is influenced by local topography.

It is important to mention that the results shown in Fig. 12 (i.e. spatial variability of strong motion) do not include the site-specific site amplification. Even when the site parameter, such as the average shear-wave velocity, is identical, it is well-known that the local site effects vary significantly. On the other hand, the local topographical effects on tsunami waves are included in the tsunami simulation results shown in Fig. 13, thus exhibiting the stronger spatial dependency of the tsunami hazard parameters, in comparison with that of the strong motion hazard parameters. Further investigations are needed to evaluate the combined effects of local site-specific characteristics on the earthquake–tsunami hazard processes.

## Conclusions

The newly developed computational framework for coupled simulation of strong motion and tsunami due to mega-thrust subduction earthquakes has great potential to address key challenges related to cascading multi-hazards risk assessments of buildings and infrastructure in the coastal region. The methodology is innovative in several aspects: (i) common physical characteristics of earthquake rupture are explicitly modeled and cascading hazard processes are simulated following the trigger phenomenon; (ii) uncertainties of source characteristics are taken into account; and (iii) both spatial and temporal hazard processes can be evaluated. A demonstration of the stochastic coupled simulation of strong motion and tsunami was presented for the case study of the 2011 Tohoku earthquake from retrospective viewpoints. The framework provides new opportunities to assess the dependency between strong motion and tsunami predictions due to common uncertain earthquake slip at different spatial as well as temporal scales. These have important implications on risk assessment and mitigation methods for coastal structures subjected to cascading multi-hazards.

One of the future extensions of the proposed methodology is to integrate it into probabilistic earthquake–tsunami multi-hazard analysis for mega-thrust subduction earthquakes (De Risi and Goda 2016). This requires the consideration of occurrence of such large events, which are known to be highly uncertain and are not well constrained by data (Kagan and Jackson 2013). New insights from the state-of-the-art statistical methods for predicting the occurrence of large earthquakes (e.g. Fierro and Leiva 2017) are useful for investigating the impact of uncertainties of earthquake occurrence to the overall assessment of earthquake–tsunami hazard and risk.

## References

[CR1] Allmann BP, Shearer PM (2009). Global variations of stress drop for moderate to large earthquakes. J Geophys Res.

[CR2] Ammon CJ, Lay T, Kanamori H, Cleveland M (2011). A rupture model of the 2011 off the Pacific coast of Tohoku earthquake. Earth Planets Space.

[CR3] Asano K, Iwata T (2012). Source model for strong ground motion generation in 0.1-10 Hz during the 2011 Tohoku earthquake. Earth Planets Space.

[CR4] Boatwright J (1988). The seismic radiation from composite models of faulting. Bull Seismol Soc Am.

[CR5] Boore DM (2003). Simulation of ground motion using the stochastic method. Pure Appl Geophys.

[CR6] Boore DM (2009). Comparing stochastic point-source and finite-source ground-motion simulations: SMSIM and EXSIM. Bull Seismol Soc Am.

[CR7] Central Disaster Management Council (2012) Working group report on mega-thrust earthquake models for the Nankai Trough, Japan: fault models for strong motion. Cabinet Office of the Japanese Government. http://www.bousai.go.jp/jishin/nankai/nankaitrough_info.html

[CR8] Daniell J, Khazai B, Wenzel F, Vervaeck A (2011). The CATDAT damaging earthquakes database. Nat Hazards Earth Syst Sci.

[CR9] De Risi R, Goda K (2016). Probabilistic earthquake-tsunami multi-hazard analysis: application to the Tohoku region, Japan. Front Built Environ.

[CR10] Federal Emergency Management Agency (2008) Guidelines for design of structures for vertical evacuation from tsunamis. FEMA P646, Washington, DC

[CR11] Fierro R, Leiva V (2017). A stochastic methodology for risk assessment of a large earthquake when a long time has elapsed. Stoch Environ Res Risk Assess.

[CR12] Fraser S, Pomonis A, Raby A, Goda K, Chian SC, Macabuag J, Offord M, Saito K, Sammonds P (2013). Tsunami damage to coastal defences and buildings in the March 11th 2011 *M*_w_9.0 Great East Japan earthquake and tsunami. Bull Earthq Eng.

[CR13] Fujii Y, Satake K, Sakai S, Shinohara S, Kanazawa T (2011). Tsunami source of the 2011 off the Pacific coast of Tohoku earthquake. Earth Planets Space.

[CR14] Ghofrani H, Atkinson GM, Goda K, Assatourians K (2013). Stochastic finite-fault simulations of the 2011 Tohoku, Japan, earthquake. Bull Seismol Soc Am.

[CR15] Goda K, Song J (2016). Uncertainty modeling and visualization for tsunami hazard and risk mapping: a case study for the 2011 Tohoku earthquake. Stoch Environ Res Risk Assess.

[CR16] Goda K, Pomonis A, Chian SC, Offord M, Saito K, Sammonds P, Fraser S, Raby A, Macabuag J (2013). Ground motion characteristics and shaking damage of the 11th March 2011 *M*_w_9.0 Great East Japan earthquake. Bull Earthq Eng.

[CR17] Goda K, Mai PM, Yasuda T, Mori N (2014). Sensitivity of tsunami wave profiles and inundation simulations to earthquake slip and fault geometry for the 2011 Tohoku earthquake. Earth Planets Space.

[CR18] Goda K, Kurahashi S, Ghofrani H, Atkinson GM, Irikura K (2015). Nonlinear response potential of real versus simulated ground motions for the 11th March 2011 Great East Japan earthquake. Earthq Spectra.

[CR19] Goda K, Yasuda T, Mori N, Mai PM (2015). Variability of tsunami inundation footprints considering stochastic scenarios based on a single rupture model: application to the 2011 Tohoku earthquake. J Geophys Res Oceans.

[CR20] Goto C, Ogawa Y, Shuto N, Imamura F (1997) Numerical method of tsunami simulation with the leap-frog scheme. IOC Manual, UNESCO, No. 35, Paris

[CR21] Gusman AR, Tanioka Y, Sakai S, Tsushima H (2012). Source model of the great 2011 Tohoku earthquake estimated from tsunami waveforms and crustal deformation data. Earth Planet Sci Lett.

[CR22] Hayes GP (2011). Rapid source characterization of the 2011 *M*_w_ 9.0 off the Pacific coast of Tohoku earthquake. Earth Planets Space.

[CR23] Iinuma T, Ohzono M, Ohta Y, Miura S (2011). Coseismic slip distribution of the 2011 off the Pacific coast of Tohoku earthquake (M9.0) estimated based on GPS data—was the asperity in Miyagi-oki ruptured?. Earth Planets Space.

[CR24] Iinuma T, Hino R, Kido M, Inazu D, Osada Y, Ito Y, Ohzono M, Tsushima H, Suzuki S, Fujimoto H, Miura S (2012). Coseismic slip distribution of the 2011 off the Pacific coast of Tohoku earthquake (M9.0) refined by means of seafloor geodetic data. J Geophys Res.

[CR25] Irikura K, Miyake H (2011). Recipe for predicting strong ground motion from crustal earthquake scenarios. Pure Appl Geophys.

[CR26] Japan Society of Civil Engineers (2002) Tsunami assessment method for nuclear power plants in Japan. https://www.jsce.or.jp/committee/ceofnp/Tsunami/eng/JSCE_Tsunami_060519.pdf

[CR27] Kagan YY (2017). Worldwide earthquake forecasts. Stoch Environ Res Risk Assess.

[CR28] Kagan Y, Jackson DD (2013). Tohoku earthquake: a surprise?. Bull Seismol Soc Am.

[CR29] Kawai H, Satoh M, Kawaguchi K, Seki K (2013). Characteristics of the 2011 Tohoku tsunami waveform acquired around Japan by NOWPHAS equipment. Coast Eng J.

[CR30] Kirby JT, Shi F, Tehranirad B, Harris JC, Grilli ST (2013). Dispersive tsunami waves in the ocean: model equations and sensitivity to dispersion and Coriolis effects. Ocean Model.

[CR31] Kurahashi S, Iikura K (2013). Short-period source model of the 2011 *M*_w_ 9.0 off the Pacific coast of Tohoku earthquake. Bull Seismol Soc Am.

[CR32] Kurahashi S, Irikura K (2011). Source model for generating strong ground motions during the 2011 off the Pacific coast of Tohoku earthquake. Earth Planets Space.

[CR34] Løvholt F, Kaiser G, Glimsdal S, Scheele L, Harbitz CB, Pedersen G (2012). Modeling propagation and inundation of the 11 March 2011 Tohoku tsunami. Nat Hazards Earth Syst Sci.

[CR35] Maeda T, Furumura T, Noguchi S, Takemura S, Sakai S, Shinohara M, Iwai K, Lee SJ (2013). Seismic- and tsunami-wave propagation of the 2011 off the Pacific coast of Tohoku earthquake as inferred from the tsunami-coupled finite-difference simulation. Bull Seismol Soc Am.

[CR36] Mai PM, Beroza GC (2002). A spatial random field model to characterize complexity in earthquake slip. J Geophys Res Solid Earth.

[CR37] Mori N, Takahashi T, Yasuda T, Yanagisawa H (2011). Survey of 2011 Tohoku earthquake tsunami inundation and run-up. Geophys Res Lett.

[CR38] Morikawa N, Senna S, Hayakawa Y, Fujiwara H (2011). Shaking maps for scenario earthquakes by applying the upgraded version of the strong motion prediction method “recipe”. Pure Appl Geophys.

[CR39] Motazedian D, Atkinson GM (2005). Stochastic finite-fault modeling based on a dynamic corner frequency. Bull Seismol Soc Am.

[CR40] Murata S, Imamura F, Katoh K, Kawata Y, Takahashi S, Takayama T (2010). Tsunami: to survive from tsunami.

[CR41] Okada Y (1985). Surface deformation due to shear and tensile faults in a half-space. Bull Seismol Soc Am.

[CR42] Pardo-Iguzquiza E, Chica-Olmo M (1993). The Fourier integral method: an efficient spectral method for simulation of random fields. Math Geol.

[CR43] Petrone C, Rossetto T, Goda K, Eames I (2016) Tsunami fragility curves of a RC structure through different analytical methods. In: Proceedings of the 1st International Conference on Natural Hazards Infrastructure, Chania, Paper 078

[CR44] Razafindrakoto HNT, Mai PM, Genton MG, Zhang L, Thingbaijam KKS (2015). Quantifying variability in earthquake rupture models using multidimensional scaling: application to the 2011 Tohoku earthquake. Geophys J Int.

[CR45] Satake K, Fujii Y, Harada T, Namegaya Y (2013). Time and space distribution of coseismic slip of the 2011 Tohoku earthquake as inferred from tsunami waveform data. Bull Seismol Soc Am.

[CR46] Shao G, Li X, Ji C, Maeda T (2011). Focal mechanism and slip history of the 2011 *M*_w_ 9.1 off the Pacific coast of Tohoku earthquake, constrained with teleseismic body and surface waves. Earth Planet Space.

[CR47] Somerville PG, Irikura K, Graves R, Sawada S, Wald DJ, Abrahamson NA, Iwasaki Y, Kagawa T, Smith N, Kowada A (1999). Characterizing crustal earthquake slip models for the prediction of strong ground motion. Seismol Res Lett.

[CR48] Stein S, Wysession M (2003). An introduction to seismology: earthquakes and earth structure.

[CR49] Tanioka Y, Satake K (1996). Tsunami generation by horizontal displacement of ocean bottom. Geophys Res Lett.

[CR50] Yamazaki Y, Lay T, Cheung KF, Yue H, Kanamori H (2011). Modeling near-field tsunami observations to improve finite-fault slip models for the 11 March 2011 Tohoku earthquake. Geophys Res Lett.

[CR51] Ye L, Lay T, Kanamori H, Rivera L (2016). Rupture characteristics of major and great (*M*_w_ ≥ 7.0) megathrust earthquakes from 1990-2015: I. source parameter scaling relationships. J Geophys Res Solid Earth.

[CR52] Yokota Y, Koketsu K, Fujii Y, Satake K, Sakai S, Shinohara M, Kanazawa T (2011). Joint inversion of strong motion, teleseismic, geodetic, and tsunami datasets for the rupture process of the 2011 Tohoku earthquake. Geophys Res Lett.

